# Home‐to‐School Contact and Its Impact on Students' School Belonging: A Triadic, Mixed‐Methods Approach

**DOI:** 10.1002/jcop.70066

**Published:** 2025-12-02

**Authors:** Mădălina A. Paizan, Lara Aumann, Peter F. Titzmann

**Affiliations:** ^1^ University of Mannheim Mannheim Germany; ^2^ Leibniz University Hannover Hannover Germany

**Keywords:** educational disparities, home‐to‐school contact, mixed‐methods, multilingual families, neighborhood SES, school belonging, school values

## Abstract

Home‐to‐school contact can reduce educational inequity. Yet, little research has examined how schools in low‐ and high‐SES neighborhoods engage only‐German‐speaking and multilingual families. Our mixed‐methods study addressed this gap by: (1) analyzing diversity‐related codes of conduct and communication strategies, (2) comparing teachers', parents', and students' home‐to‐school contact perspectives (MANOVA), and (3) linking them to school belonging. Participants included 944 students (*M*_age_ = 13.4; 64% multilingual), 28 classroom‐teachers (*M*_age_ = 46.8), and 352 parents (*M*_age_ = 44.5; 40% multilingual). A content analysis of school websites revealed similar diversity‐related codes of conduct, focused on tolerance and inclusion. High‐SES neighborhoods displayed more customized home‐to‐school communication strategies. Teachers and parents reported more contact in only‐German‐speaking families, and teachers reported more contact with multilingual families in high‐SES neighborhoods. All three contact perspectives predicted school belonging in high‐SES neighborhoods, but only teacher and student‐reported contact showed effects in low‐SES neighborhoods. Findings call for disentangling informant, SES, and migration factors in diverse schools.

## 1 Introduction

According to the Matthew Effect, wherein students with greater initial resources accumulate more advantages over time (Pfost et al. 2014), wealthy students tend to access more learning resources and opportunities compared to their less privileged peers. A parallel can be observed in the experiences of ethnic majority in comparison to ethnic minority students, not only because of a lack of resources, but also through additional language barriers. As a result, systemic educational disparities pervade today's schools. A central element to bridge these gaps is increased home‐to‐school contact, which can improve home–school interactions and a mutual exchange of students' needs and opportunities (Hill and Tyson 2009). Frequent and supportive home‐to‐school contact, however, is not a naturally occurring phenomenon. Instead, it depends on structural opportunities of how schools officially organize the contact, and individual perceptions of the quality and quantity of mutual exchange. In addition, the structural and individual levels of home‐to‐school contact often differ depending on resources (the socioeconomic standing of the neighborhood) and language barriers (the family language may not be the language of the majority). How these factors are intertwined and how these factors can explain the role of home‐to‐school contact in students' school adaptation is, however, not fully understood.

This multi‐informant mixed‐methods study aims to deepen our understanding of how schools can support the involvement of parents in school activities by examining two aspects of home‐to‐school contact: structural opportunities and individual perceptions. Structurally, we analyze diversity‐related codes of conduct and communication strategies of schools in low and high socioeconomic status (SES) neighborhoods to assess the opportunities available for teacher‐parent contact. The diversity‐related code of conduct is an important part of a school's equitable leadership and a first pre‐requisite to enable action and collaboration with families and improve students' outcomes (Leithwood 2021; Moral et al. 2020). The diversity‐related code of conduct represents a structural opportunity for home‐to‐school contact because it includes core values and principles, which guide school leaders, educators, and all other school members. As schools and families are becoming more diverse, adopting a diversity‐related code of conduct is a first step forward because it encourages adopting culturally sensitive ways to involve families. For example, a school whose core value is to create intercultural interactions may be more inclined to develop flexible activity formats so that parents and teachers come together. Next to the diversity‐related code of conduct, communication strategies adopted by a school are a structural opportunity for home‐to‐school contact because they can ease and facilitate the frequency of contact. For example, if various methods of communication (e.g., starting at different hours, working one‐to‐one or in larger teams of parents and teachers) are used, the probability of a high participation from parents increases. On the individual level, we examined the frequency of contact from parents' and teachers' perspectives and the quality of contact between parents and teachers from students' perspectives. The frequency of contact is an assessment of opportunities for mutual exchange and offers a more objective view of the relationship between parents and teachers. Research has generally shown that when teachers and parents constantly communicate with each other (i.e., contact frequency), students show higher levels of school belonging, school engagement, and obtain better academic results (Topor et al. 2010). The students' perspective gives insight into the perceived quality of contact between parents and teachers, which has been found to be critical for student outcomes (Garbacz et al. 2021; Lasater 2016). For example, when students view parents and teachers to maintain an open, respectful communication, they tend to show improved school behavior (e.g., following school rules) and increased engagement, as they feel supported and motivated both at home and at school. Hence, we assessed contact quality among students and contact frequency among parents and teachers, as these focus on different aspects of the relationships between teachers, students, and parents, yet work toward similar outcomes.

To achieve these aims, we conducted a qualitative analysis of the diversity‐related code of conduct and communication strategies among schools in neighborhoods of varying SES to understand structural opportunities for home‐to‐school contact. Additionally, we used multi‐informant questionnaires to quantitatively investigate teachers', parents', and students' perspectives of home‐to‐school contact, focusing on the effects of SES and family language as indicators of migration background. Finally, we analyzed how home‐to‐school contact, in conjunction with family language, correlates with students' sense of school belonging across low‐ and high‐SES neighborhoods. By integrating these facets, this study offers a comprehensive view of the barriers and enablers of home‐to‐school contact, with implications for reducing educational disparities through diversity‐based school practices.

### 1.1 Home‐to‐School Contact

Drawing on Bronfenbrenner's bioecological model (1979), this study looks at how students' academic development is influenced by the interaction of multiple interconnected systems. Especially microsystems—such as the family, peer group, school, neighborhood, and religious affiliations—play a direct and immediate role in shaping an individual's growth. These microsystems also constantly interact and form new contexts of development called mesosystems, such as home‐to‐school interactions or neighborhood‐school interactions. In this study, we focus specifically on how the extended mesosystem home–school‐neighborhood impacts students' development. We examine how the socioeconomic position of neighborhoods shapes the frequency and quality of home‐to‐school contact, which in turn influences students' sense of school belonging, a critical outcome for positive academic development.

Regarding home‐to‐school contact, a steady and trustworthy interaction between parents and teachers does benefit students' learning and social adjustment to school, for example, through a timely flow of information that enables adequate support (Garbacz et al. 2018; Smith et al. 2020). In this interaction, home‐to‐school contact is particularly fruitful if it involves a bidirectional exchange of information between parents and teachers (Epstein and Sanders 2002). Home‐to‐school contact, however, does not follow a linear trajectory from kindergarten to high school but varies depending on age, transition phase, and students' needs. For instance, the contact between parents and teachers is more intense when children transition from preschool to primary school, as there are numerous structural changes and behavioral expectations from the school system that need to be learned (e.g., classroom rules; E. Murray et al. 2015). Furthermore, it intensifies when students show additional learning difficulties (e.g., reading and spelling difficulties, ADHD; D. W. Murray et al. 2008).

During middle school, parents typically take on a more passive role, participating less in school activities and rather focusing on monitoring children's grades (Thomas et al. 2020). At the same time, academic demands increase, and questions about youths' educational future and career path gain in importance. During this phase, home‐to‐school contact is crucial to support adolescents in navigating the complexity of the school system (e.g., various expectations from multiple teachers; selecting a practical training) and coping with academic challenges. Moreover, teacher–parent contact has proven to be an important predictor of home‐based parental involvement among adolescents (Paizan et al. 2022). Therefore, teacher–parent contact does not lose importance during adolescence. On the contrary, it remains an essential marker of students' thriving, as both parents and teachers support students in managing school‐related and self‐regulation tasks, though the nature and content of interactions evolve over time.

Despite the benefits of an increased home‐to‐school contact, the level can vary substantially, depending on both the structural level (e.g., school guidelines for teachers on how to engage with parents, school projects) and the individual level (e.g., how often do parents and teachers communicate with each other, how do students perceive this contact). On the structural level, home‐to‐school contact implies that “schools create opportunities for teachers and parents to interact” (Serpell and Mashburn 2012). Thus, the structural level refers to school‐initiated opportunities that facilitate teacher‐parent interactions, such as school policies, digital communication platforms, or events aimed at parental engagement. These structures aim to make it easier for families to connect with the school, though they may not be equally accessible to all, especially when language and socioeconomic disparities come into play (Serpell and Mashburn 2012). Typical modes of home‐to‐school contact on a structural level can either be digital (e.g., school websites, school e‐mail; Piller et al. 2023) or analog (parent‐teacher conferences, volunteering, or being a member of the Parent Teacher Association; Gibbs et al. 2021; Munthe and Westergård 2023). However, these types of home‐to‐school contact may be somehow restrictive because they limit the engagement of some parents (e.g., monolingual habitus of the school; limited number of parents who can adopt a particular role in the Parents' Association; work time collides with volunteering hours). Notably, a welcoming school environment and informative communication strongly predicted parents' school‐based involvement (Park and Holloway 2018). Research on the structural level of home‐to‐school contact is, however, scarce and does not investigate neighborhood‐specific contact strategies of schools.

Besides the structural level, with focus on the school as a whole, there is also an individual level, because the home‐to‐school contact, that is, the frequency of direct interactions between parents and teachers and how the parent‐teacher contact quality is perceived by students often varies within the same school.

Structural and individual levels on home‐to‐school contact, while interrelated, address different aspects of the home‐to‐school dynamic and require separate examinations to understand how they contribute to home‐to‐school contact and, eventually, to educational outcomes. For families from diverse backgrounds, including variations in SES and linguistic backgrounds, the structural and individual levels of home‐to‐school contact are influenced by distinct challenges and opportunities. When SES is studied, research has shown that migration history often is a confounding variable, although SES usually shows stronger effects for students' outcomes than migration history (Schofield 2006; Oppermann and Lazarides 2023). Our study aimed at separately looking at these two social status characteristics and examined how they relate to the contact between schools and families. Migration history can be defined in different ways depending on the research of interest: length of residence, citizenship, ethnic identity, or language. In this paper, we define migration background through the family language, because (majority) language skills are mostly mandatory to achieve home‐to‐school contact in Germany. Neighborhood SES is defined by means of the rent charges and population characteristics in the specific area. Strengthening home‐to‐school contact emerges as a promising approach for fostering equity, as it can not only support academic success across varied socioeconomic and linguistic backgrounds but can also play a foundational role in mitigating educational inequity (Barnard 2004). However, so far, elaborated studies on home‐to‐school contact are missing (Park and Holloway 2018).

### 1.2 Home‐to‐School Contact and Neighborhood SES

Students' experiences in school and educational disparities are often closely associated with students' socioeconomic backgrounds (Bronfenbrenner 1979). The SES impacts access to resources, opportunities, and school options, which in turn affects the nature of contact between teachers and parents in school‐related matters (Skopek and Passaretta 2021). Across the EU, nearly 25% of children and adolescents are at risk of poverty (Statistisches Bundesamt 2024), and students from affluent families are reportedly 3.4 times more likely to receive recommendations for higher education compared to students from low‐income backgrounds with similar academic performance (Hußmann et al. 2017). These disparities begin at home, even before children enter the school system, and widen throughout primary and secondary education, which does increase the gap between high and low SES students over the educational course (Skopek and Passaretta 2021). Families' financial circumstances typically determine their place of residence, which can lead to residential segregation and thus create neighborhood‐based differences in school resources and student body composition. The resulting segregation further entrenches educational inequities and impacts parents' involvement in school and students' development.

At the structural level, home‐to‐school contact can be substantially and particularly impacted by neighborhood‐related aspects (Desforges and Abouchaar 2003). In high SES neighborhoods, schools tend to have access to more resources, stronger social networks, and often better‐resourced communication structures that support home‐to‐school contact (Desforges and Abouchaar 2003). On the contrary, low SES neighborhoods are more often characterized by economic stress, fewer resources, and weaker social networks, which can negatively affect the home‐to‐school contact (Waanders et al. 2007; Sime and Sheridan 2014). Moreover, typical structured engagement opportunities (i.e., parent‐teacher meetings, workshops, or volunteering programs) may sometimes inadvertently exclude parents with inflexible working hours or those unfamiliar with certain school expectations, which is often the case in low SES neighborhoods (Kohl et al. 2013). In sum, there is a need to identify neighborhood‐specific needs and opportunities for home‐to‐school contact, particularly by differentiating how schools engage parents in schools from low‐ and high‐SES neighborhoods.

On the individual level, parents from low SES backgrounds may face contact barriers, such as time constraints, job‐related pressures, and the system itself may not always be adequately structured to be accessible or responsive to their diverse needs and experiences. Moreover, the likelihood of a socio‐cultural mismatch between parents' and teachers' views in low SES neighborhoods is often higher. Whereas parents in such neighborhoods may have less direct experience with the school system and report missing information about the school life, the teachers are often less aware of the home realities and struggles of low SES families, such as inflexible working hours, long shifts, and childcare duties that cannot be delegated (Kohl et al. 2013; Schneider and Arnot 2018). This may be one reason why some school strategies to improve contact with parents in lower SES neighborhoods fail to reach all parents. Teachers in low SES neighborhoods often report challenges in reaching parents who may feel alienated by formal school structures or perceive the school environment as unaccommodating of their needs (Schneider and Arnot 2018). Thus, there is a heightened need for schools to recognize the specific contact needs and limitations of low SES families, which, if addressed, could improve the frequency of parent‐teacher interactions.

### 1.3 Home‐to‐School Contact and Multilingual Families

Beyond SES, migration history often plays a significant role in shaping parent‐school relationships and students' educational success. Language barriers of parents, in particular, create additional challenges for engaging with schools, leading to a reduced ability to support their children's education effectively. The latest German National Education Report (2024) highlights the compounded challenges faced by first‐ and second‐generation immigrant students, where 60% are affected by education‐related risk factors—such as low parental education, financial strain, and language barriers—compared to only 20% of nonimmigrant students. Language skills are essential for all types of parental involvement, whether that involves supporting children's learning at home (home‐based parental involvement), understanding school requirements (academic socialization), or communicating directly with teachers (school‐based parental involvement) (Hébert et al. 2024). This study employs the terms “multilingual” and “only‐German‐speaking families” to delineate the linguistic profiles of families. It defines multilingualism as the use of multiple languages in diverse contexts, including public and school settings as well as private and family domains. It further delineates only‐German‐speaking families as those in which German is the sole language used by the family in all contexts. For multilingual families, imperfect proficiency in the dominant school language can restrict their ability to fully participate in school‐related activities and to engage effectively with teachers, resulting in reduced home‐to‐school contact and limited access to school resources that facilitate learning. Research has shown, for example, that minority adolescents frequently act as language brokers for their parents—also when they are second‐generation immigrants (Cline et al. 2014), which shows parental insecurity in parent‐school communication. Language barriers can be associated with missing knowledge about the school system and different expectations identified as consequences (Hébert et al. 2024; Kohl et al. 2013; Schneider and Arnot 2018).

On the structural level, schools can either facilitate or hinder contact by providing support for multilingual families. Superficial forms of home‐to‐school contact (e.g., international meals), however, have been found to be less successful than an investment in long‐term, official school structures for parental school participation (e.g., school councils; Díez et al. 2011), probably also because they reinforce stereotypic images of some groups. In contrast, schools that emphasize values of diversity and cultural pluralism are more likely to adopt policies that make contact accessible for diverse family communities (Haines et al. 2015). A positive and inviting school vision of language diversity may help create a welcoming environment for multilingual parents and encourage engagement with school life (McWayne et al. 2022). For example, some schools may translate key documents into commonly spoken languages or provide interpreters for parent‐teacher meetings, while others may overlook such needs, reducing opportunities for multilingual families in the long term (Coady and Ankeny 2019; Ulbricht et al. 2022). Without this accommodation, parents with limited language skills may struggle to engage fully, which could lead to misunderstandings or feelings of exclusion. Schools in Germany follow public guidelines regarding their work with families (KMK 2018), but they also have the possibility to implement their own parent programs. Therefore, our goal was to study how schools understand diversity and examine which specific lines of action schools adopt to promote home‐to‐school contact.

At the individual level, language barriers also impact direct interactions between parents and teachers. Parents who do not speak the dominant language fluently may hesitate to reach out to teachers, or they may avoid school functions altogether, limiting opportunities for building trust and collaboration (Kohl et al. 2013). Conversely, teachers may feel unprepared to communicate with parents who have limited proficiency in the school language, which can lead to a reliance on superficial interactions rather than meaningful engagement.

Thus, both structural accommodations, such as translation services, and targeted teacher‐parent contact efforts are essential for creating equitable home‐to‐school contact for multilingual families. Nevertheless, there has been little investigation of how schools address the home‐to‐school contact among multilingual families to make it accessible for non‐German speaking parents, how parents and teachers communicate with each other and how the parent‐teacher contact may improve students' school belonging.

### 1.4 Home‐to‐School Contact and School Belonging

A key assumption for this study is that effective home‐to‐school contact can enhance students' sense of school belonging, which refers to the feeling of being accepted, respected, included, and supported within the school environment (Goodenow 1993). School belonging has been shown to correlate with a range of positive outcomes, including motivation, social‐emotional well‐being, and academic success (Tillery et al. 2013; Allen et al. 2018; Korpershoek et al. 2020). Thus, school belonging serves as an important marker of educational equity and student well‐being, especially in diverse socioeconomic and linguistic contexts. Therefore, in this study, we investigate school belonging from students' perspective as a marker for reducing educational disparities.

Research has identified several factors that foster school belonging, including peer support, positive teacher‐student relationships, and parental involvement (Uslu and Gizir 2017; Ahmadi et al. 2020). However, prior studies have typically investigated these factors in isolation—either from the perspective of teachers or parents alone, overlooking the combined influence of both on students' school belonging (Haines et al. 2015). In addition, teachers, students, and parents have been shown to report contrasting perceptions of various school concepts (Paizan et al. 2024; Schneider and Arnot 2018). Hence, in this study, we address this research gap and aim to examine how different perspectives (teacher vs. parents vs. students) on home‐to‐school contact contribute to student's sense of belonging, and how these dynamics may differ across schools in low‐ and high‐SES neighborhoods.

## 2 Current Study

In this mixed‐methods study, we employ an embedded‐correlational design to gain a more comprehensive understanding of the structural and individual levels of home‐to‐school contact, with the qualitative component embedded within the larger quantitative framework (Creswell and Clark 2017). Data collection occurred in two distinct study phases, involving two independent strands: a quantitative survey and qualitative closed‐ended questions. The quantitative data comprises self‐reported ratings from students, parents, and teachers, while the qualitative data includes organizational characteristics and website information, coded into two main categories. Each data set was analyzed separately, and the findings were integrated into a joint discussion.

The overall purpose was to comparatively examine home‐to‐school contact in seven integrated comprehensive schools from low and high SES neighborhoods in Germany and investigate relations of home‐to‐school contact with students' school belonging, a well‐known indicator of educational success. The qualitative part focused on the first research aim (structural level of home‐to‐school contact) and investigated the structural opportunities of schools in low and high SES neighborhoods, including diversity‐related code of conduct and communication strategies to involve parents in the school life (RQ1). The quantitative part focused on the second research aim (individual level of home‐to‐school contact) and investigated teachers', parents' and students' perspectives on home‐to‐school contact. Moreover, it targeted to examine how these individual perspectives relate to students' sense of school belonging in low and high SES neighborhoods. Based on theoretical considerations (e.g., higher similarity between high SES families and teachers' views), we expected more frequent contact between parents and teachers in high SES as compared to low SES neighborhoods (H1). Since language skills are a key factor for home‐to‐school contact in (German) schools, we expected a more frequent home‐to‐school contact with only‐German‐speaking families in comparison to multilingual families (H2). In addition, considering the long‐term benefits of the home‐to‐school contact on students' outcomes (Vaz et al. 2015; Smith et al. 2019), we expected home‐to‐school contact (rated from all three actors) to be positively related to students' school belonging (H3).

## 3 Materials and Methods

All scales used in the study are validated and internationally approved. Although all students reported very good German language skills and were enrolled in German schools, it was tested for measurement equivalence to eliminate cultural/contextual differences in the understanding of items. The parental questionnaire was administered in German as the main language and translated in five other most spoken languages for our study's area: English, Turkish, Russian, Polish and Arabic. However, only 23 out of 352 parents filled in questionnaires that were not in German (most of them were Turkish). Analyzes among youth from only‐German‐speaking and multilingual families by using exploratory factor analyzes and congruence coefficient Tucker's Phi (van de Vijver and Poortinga 2002) showed a very good fit for all scales (φ ≥ 0.99). This ensured the validity and reliability of the scales across diverse cultural backgrounds within the sample.

### 3.1 Sampling and Participants

The data used in this study originate from a larger German project on the role of parent–teacher interactions in improving academic achievement of ethnic minority and majority adolescents (“LEITER”). Adolescents were recruited from integrated comprehensive schools (Integrierte Gesamtschule; IGS). IGS are state schools that offer vocational and higher education tracks to students of all competence levels without any fees or entry exams. These schools typically serve families from the local neighborhood, who share similar socioeconomic backgrounds (Baumert et al. 2003). The sample used in this study was drawn from schools with high ethnic and cultural diversity (13.5% ethnic minorities compared to 5.2% in other school types) in and around Hanover, Germany. Hanover is a multicultural city with 41.4% of its population reporting family migration history and 52% of its children under 18 years holding dual citizenship (i.e., German citizenship and another citizenship; Landeshauptstadt Hannover 2024). Data were collected from 944 students from grades 6th to 10th (*M*_age_ = 13.4, SD = 1.4, 48% female, 64% from multilingual families) from seven schools in Hanover, their 28 classroom‐teachers (*M*_age_ = 46.8, SD = 10.7, 74% female) and 352 parents (*M*_age_ = 44.5, SD = 6.4, 84% female, 40% multilingual). Multilingual parents (*M* = 3.58, SD = 0.04) showed significantly lower levels of German language skills than only‐German‐speaking families (*M* = 3.96, SD = 0.03), *F*(1, 243) = 53.43, *p* < 0.001, *η_p_* ^2^ = 0.18, even if controlled for family SES; which had no effect: *F*(1, 243) = 0.039, *p* = 0.84, *η_p_* ^2^ = 0.00. School‐level demographic data were obtained from school principals and school websites.

### 3.2 Measures

#### 3.2.1 Frequency of Home‐to‐School Contact (Teachers' and Parents' Perspective)

The frequency of home‐to‐school contact was measured with a scale developed by Serpell and Mashburn (2012). This construct captured the frequency of different types of contact, such as phone contacts, voluntary contacts and parent‐teacher conferences. The scale comprised six items assessed from 1 to 7, where 1 means *never* and 7 *always*. The internal consistency was *α* = 0.70 for parents and α = 0.74 for teachers.

#### 3.2.2 Quality of Home‐to‐School Contact (Students' Perception)

Students were asked how positive they perceive the contact between their parents and their teachers on a self‐developed item, with a scaling from 1 = *(rather) negative* to 7 = *(rather) positive*.

#### 3.2.3 School Belonging

Students' school belonging was measured with the “Psychological Sense of School Membership Scale (PSSM)” by Goodenow (1993). The scale comprised two items assessed from 1 to 7, where 1 means *not applicable at all* and 7 means *fully applicable*. The internal consistency was *α* = 0.79.

#### 3.2.4 Family Language

Students were asked if they speak a language other than German at home by answering *yes* or *no* and mentioning the spoken language(s) if this was the case.

### 3.3 Socioeconomic Status (SES)

The financial situation of the family and the socioeconomic living context (low vs. high) of the students were assessed based on the average rent in the neighborhood. The information on the average rents of the neighborhoods is based on government data and was compared with the information provided by the youths and their parents on net income and financial situation.

### 3.4 Study Procedure

The University's Ethics Committee, the regional school authority, and school directors gave their consent to conduct the study in schools. All participants were informed about the study's purpose, voluntary participation, and the means of withdrawal at any time without any consequences. Students filled in the questionnaires in the classroom after receiving consent from their parents, while teachers and school principals were given the opportunity to either fill in the questionnaires in the classroom or send them back by post. Parents were asked through their children to fill in the questionnaires at home and send them back by post. Teachers reported on the contact frequency to one parent of each individual child in their classroom and parents reported on the contact frequency to the classroom teacher. All participants completed paper–pencil questionnaires between June and October 2019 in the classroom accompanied by a team of researchers with multicultural heritage. Students and parents were offered a 10€ voucher for participation.

### 3.5 Data Analysis

#### 3.5.1 Qualitative Part–Structural School Level

To investigate RQ1, we utilized a Qualitative Content Analysis approach based on Mayring's (Mayring 2003) content structuring methodology to systematically evaluate the structural characteristics of home‐to‐school contact of seven schools in Germany. Data were collected through a comprehensive review of the schools' official websites and questionnaires from all seven school principals. The data were transcribed so that all data is available in written form. The analysis followed a deductive approach by applying two main categories: (a) diversity‐related code of conduct—how each school defines and promotes diversity within its institutional framework and (b) communication strategies on the structural level—the strategies schools use to facilitate home‐to‐school contact, with a particular focus on families from ethnically diverse backgrounds. The following steps were carried out one after the other:

1. Determination of the material (selection and marking of website content for each school and questionnaire parts regarding the two predefined categories).
2. Extraction and coding (text data was segmented and assigned to the two categories).
3. Abstraction (each segment was analyzed to identify relevant themes and patterns that represent the schools' general approaches).
4. Interpretation (themes were interpreted in the broader context of diversity‐related code of conduct and communication strategies in schools, with an emphasis on comparisons between schools in low and high‐SES neighborhoods).

#### 3.5.2 Quantitative Part–Individual Level

This study employed SPSS 29.0 and *M*plus 8 software for data management and analysis. First, we performed descriptive statistical analysis and correlation analysis of the study variables. In a first step, to test hypotheses H1 and H2, we conducted a multivariate ANOVA with home‐to‐school contact from parental, teacher and student views as dependent variables. To test hypothesis H3, we utilized multi‐group regression analyzes.

## 4 Results

To analyze the structural characteristics facilitating or hindering home‐to‐school contact we investigated seven German schools. Table 1 presents the structural characteristics of each school separately. These schools were categorized based on their neighborhoods' SES, with three schools located in low SES neighborhoods (Schools A, B, and C) and four schools in high SES neighborhoods (Schools D, E, F, and G).

**Table 1:** Organizational characteristics of seven integrated comprehensive schools in Hanover, Germany.

	School	Participants in the LEITER‐Project	#Students‐Total^a^	% Students with immigrant descent	#Teachers‐Total^a^	% Teachers with immigrant descent	#Special needs teachers*	#Students per special needs teacher	#Students with immigrant descent per special needs teacher	Monthly Net‐Income Parents (in €)
Schools in low^b^ SES neighborhood	A	93	930	84%	111	27%	8	116	97	2000–3000
	B	330	880	75%	84	42%	13	68	51	2000–3000
	C	75	608	71%	82	15%	8	76	54	2000–3000
Schools in high^b^ SES neighborhood	D	93	1594	9%	179	6%	14	114	11	3000–4000
	E	182	1221	57%	135	11%	10	122	70	3000–4000
	F	28	500	6%	39	3%	1	500	30	3000–4000
	G	338	850	50%	80	25%	6	142	70	3000–4000

a The data were reported by the school principal.

b Low versus high SES neighborhood: measured by means of the rent charges and population markers.

The total number of students per school ranged from 500 to 1594. Schools in low SES neighborhoods reported a higher percentage of students with migration history (*M* = 70.0%, SD = 12.65) compared to schools in high SES neighborhoods (*M* = 30.3%, SD = 23.63). Similarly, the percentage of teachers with migration history was higher in schools located in low SES neighborhoods (*M* = 27.3%, SD = 11.09) compared to those in high SES areas (*M* = 8.3%, SD = 4.16). Special needs support also varied, with the number of special needs teachers per school ranging from 1 to 14. Schools in low SES neighborhoods tended to have fewer students per special needs teacher (*M* = 100.5, SD = 27.46) than schools in high SES neighborhoods (*M* = 245.3, SD = 175.12). In terms of socioeconomic background, schools in low SES neighborhoods reported lower parental net monthly incomes (between €2000 and 3000) compared to schools in high SES neighborhoods, where parental income was generally higher (between €3000 and 4000).

### 4.1 Qualitative Analysis on Structural School Level

To investigate RQ1, we used Qualitative Content Analysis. Two primary categories were explored: (1) diversity‐related code of conduct and (2) communication strategies on the structural level. In terms of diversity‐related code of conduct, the analysis revealed differences between schools in low and high SES neighborhoods (Table 2). Schools in low SES areas (e.g., Schools A and C) emphasized diversity and equal opportunities. For instance, School A portrayed itself as a student‐focused space, where students of diverse religions, nationalities, and talents could interact and learn together. School C also underscored the importance of respecting different cultures and lifestyles, promoting a school environment where diversity is seen as integral to the educational experience. For School B, there was no information available on the schools' website, and teachers and the school principal mentioned no specific information regarding home‐to‐school contact.

**Table 2:** Structural Level of Home‐to‐School contact: diversity‐related code of conduct and communication strategies.

	School	Diversity‐related code of conduct	Communication strategies
Schools in low SES neighborhood	A	A school for all children: students of different religions, nationalities and talents interact with and from each other on a daily basis.	1. Career counseling together with parents (including a parents’ evening on the topic of job orientation) 2. Introduction days with parents at the beginning of the school year (11th grade) 3. Counseling for parents (financial support, parenting courses, school support, etc.) 4. School Parents' Council
	B	No information given regarding home‐to‐school contact.	No information given regarding home‐to‐school contact
	C	Our goal is to achieve equal opportunities for each individual. Everyone respects the different cultures, religions, holidays and lifestyles.	1. Newsletter 2. Registration is possible over the phone 3. School Parents’ Council (meetings 4x per school year) 4. Parent‐teacher conferences (meetings 4x per school year) 5. Parents are encouraged to contact the main teachers/parent representatives if problems arise 6. Open house day 7. Information evenings for new students and their parents
Schools in high SES neighborhoods	D	A school for all children, that understands the diversity of all as an enriching basis for discussions in which everyone can participate and grow on an equal footing (following the recommendations of the UN‐Charta on inclusion).	1. School Parents‘ Council (meetings 2x per school year) 2. Special E‐Mail address for parents 3. School's social work has a particular focus on parents (counseling for parents; parent work, events for parents) 4. Open house day (guided tours for students and parents) 5. School program includes the developmental goal “Cooperation with people working at the school, parents and extracurricular institutions/companies will be intensified” 6. (Thematic) parent‐teacher conferences 7. Learning plans and weekly overviews are available for parents 8. Parents involved in break activities (e.g. selling snacks) 9. Parent & student consultation days
	E	School values: student empowerment, learning outside of the school, create intercultural interactions.	1. Newsletter 2. Open house day 3. The mission statement “Open school” includes the involvement of parents 4. School's social work has a particular focus on parents 5. Psychological counseling services for parents; 6. Several Parent project groups 7. School Parents' Council (meetings 4x a year) & school board 8. Parent‐teacher conferences
	F	We work together with patience, composure, tolerance and humor.	1. Newsletter 2. Counseling services for parents 3. Parent representatives 4. Open house day 5. Special complaints approach for parents
	G	We keep our students’ school paths open for a long time because the development of the individual takes place at different speeds. Other school values: responsibility for each other, code of conduct, community learning, expert teams per classroom and school year to ensure the standards of inclusion.	1. Project week including a presentation for parents; 2. Newsletter 3. School Parents' Council (2x per school year); 4. One task of the school is to support “qualified parent work” 5. Counseling of parents, events for parents 6. Parent & student consultation days 7. Registration is possible over the phone (help with filling out the registration form) 8. Online access to parents' evening presentations 9. Parent‐teacher conferences: online access to archived posts on school life

In contrast, schools in high SES neighborhoods (e.g., Schools D and G) adopted a more structured approach to diversity, often linking their values to broader educational frameworks, such as the UN‐Charta on inclusion. School D's diversity approach, for example, reflects a holistic perspective of the school system by not just focusing on students but also on the family and community. This is mirrored especially in the communication strategies with parents: thematic parent‐teacher conferences, the social worker in the school organizes projects specifically for parents' needs and, students' learning progress is available for students and parents.

Regarding communication strategies, the analysis showed noteworthy differences in how schools facilitated home‐to‐school contact. In low SES neighborhoods, communication strategies were designed to be accessible and supportive. For instance, School A provided career counseling sessions involving parents and offered various forms of parental support, such as financial aid and parenting courses. School C also engaged parents through parent‐teacher conferences, and regular updates on the school website, reflecting an understanding of the diverse needs of their parent populations.

Schools in high SES neighborhoods (e.g., Schools D and E), on the other hand, employed more robust and frequent (2 to 4 parent conferences per year) communication strategies tailored to their parent communities. School D provided a dedicated email address for parents, organized thematic parent‐teacher conferences, and facilitated regular school council meetings, while school F created a special complaint approach for all school parties involved. These schools also offered opportunities for parents to be actively involved in school life, such as selling snacks during breaks or participating in school social work. This proactive approach suggested that parents were not only seen as passive recipients but also active participants within the school.

Generally, all schools shared a commitment to promoting diversity, with a common emphasis on engaging parents through structured communication strategies and activities. All schools except School B (we had no information from this school), regardless of SES, implemented regular updates on school life via their websites and organized open house days, parent‐teacher conferences, and meetings of the School Parents' Council, indicating a shared recognition of the importance of maintaining an open line of communication with parents.

### 4.2 Quantitative Analysis on Individual Level

The descriptive statistics and correlations of the study variables are shown in Table 3. The first hypothesis (H1) posited that schools in high SES neighborhoods would exhibit more frequent and positive contact between parents and teachers compared to schools in low SES neighborhoods. The analysis (MANOVA) revealed no significant differences in home‐to‐school contact from all three perspectives (see Table 4), but it is noteworthy that the effect for teachers and parents was in the expected direction. Thus, H1 was not supported.

**Table 3:** Study variables and sample characteristics.

						Correlations											
			*M*	Range	SD	1	2	3	4	5	6	7	8	9	10	11	12
Students	1	Age	13.4	12–17	1.4	1											
	2	Female gender	48%			−0.007	1										
	3	Only‐german‐speaking families	64%			0.101**	−0.053	1									
	4	Home‐to‐school contact quality	5.69	1–7	1.5	−0.090**	−0.056	−0.058	1								
	5	School belonging	4.45	1–7	1.8	−0.220**	−0.088**	−0.166**	0.281**	1							
Teachers	6	Age	46.8	29–62	10.7	0.069	0.052	−0.108*	−0.107*	−0.025	1						
	7	Female gender	84%			−0.049	−0.077	−0.092*	0.107*	0.161**	0.275**	1					
	8	Home‐to‐school contact frequency	5.05	1–7	1.4	0.070	0.020	0.407**	0.094*	−0.107*	−0.084	−0.170**	1				
Parents	9	Age	44.5	30–68	6.4	0.331**	0.021	0.274**	−0.037	−0.037	−0.095	−0.133	0.207*	1			
	10	Female gender	84%			−0.057	−0.121	0.013	−0.019	0.043	−0.014	0.079	−0.089	0.148*	1		
	11	Only‐german‐speaking families	40%			0.045	−0.041	0.740**	0.034	−0.058	−0.185*	−0.035	0.291**	0.182**	0.011	1	
	12	Home‐to‐school contact Frequency	5.89	1–7	1.1	−0.046	−0.033	0.326**	0.111	0.044	−0.109	−0.075	0.387**	.089	−0.095	0.256**	1

* *α *< 0.05.

** *α *< 0.01.

**Table 4:** Multivariate ANOVA with home‐to‐school contact as dependent variables.

		Home‐to‐School contact			Outcome
		Teacher perspective (Contact frequency)	Parental perspective (Contact frequency)	Student perspective (Contact quality)	School belonging
Low SES Neighborhoods	*M*	5.46	5.63	5.81	4.75
	SD	0.16	0.19	0.24	1.73
High SES Neighborhoods	*M*	5.74	5.84	5.63	4.19
	SD	0.10	0.12	0.15	1.78
		*F* = 1.93, *p* = 0.17, *η2* = 0.02	*F* = 0.84, *p* = 0.36, *η2* = 0.01	*F* = 0.47, *p* = 0.49, *η2* = 0.01	*F* = 21.67, *p* < 0.001, *η2* = 0.16
Multilingual Families	*M*	5.13	5.27	5.87	4,68
	SD	0.15	0.18	0.22	1,81
Only‐German‐speaking Families	*M*	6.05	6.19	5.57	4,08
	SD	0.12	0.14	0.18	1,66
		*F* = 22.79, *p* < 0.001, *η2* = 0.15	*F* = 18.74, *p* < 0.001, *η2 *= 0.12	*F* = 1.13, *p* = 0.29, *η2* = 0.01	*F* = 24.34, *p* < 0.001, *η2* = 0.17
SES × Family Language		*F* = 3.21, *p* = 0.05, *η2* = 0.05	*F* = 1.36, *p* = 0.25, *η2* = 0.01	*F* = 3.20, *p* = 0.05, *η2* = 0.05	

The second hypothesis (H2) suggested that home‐to‐school contact would be more frequent with only‐German‐speaking families compared to multilingual families. Results of the MANOVA showed significant differences across multiple perspectives (see Table 4). Teachers and parents agreed in their reports that only‐German‐speaking families had significantly more frequent contact than multilingual families (Teachers: *M* = 6.05, SD = 0.12 vs. *M* = 5.13, SD = 0.15; Parents: *M* = 6.19, SD = 0.14 vs. *M* = 5.27, SD = 0.18). However, the students' perspective on teacher–parent contact quality did not exhibit significant differences between only‐German and multilingual families. These findings largely support H2, demonstrating more frequent contact in only‐German‐speaking families from the perspectives of teachers and parents, although the different frequency was not reflected in differences in students' perception of their contact quality.

**Figure 1:**
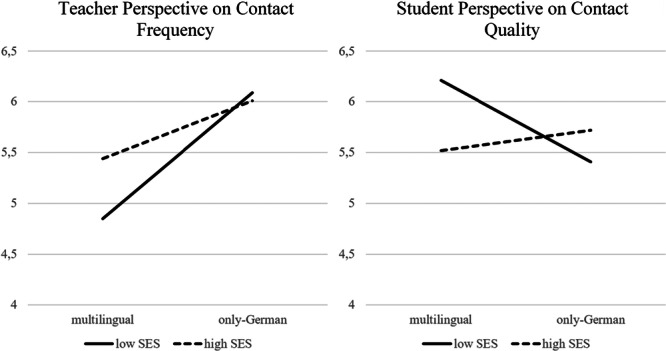
Interaction effects of SES neighborhood and family language for teachers' and students' perspective of Home‐to‐School contact.

In addition, as speaking other family languages and SES are often associated, we wanted to differentiate effects of low versus high SES neighborhood (SES neighborhood) and only‐German speaking versus multilingual families (family language), using moderation terms. Analyses revealed significant interaction effects for teachers' report on contact frequency (*F* = 3.21, *p* = 0.05) and students' perspective on the teacher‐parent contact quality (*F* = 3.20, *p* = 0.05). As illustrated in Figure 1, in only‐German speaking families no significant differences in home‐to‐school contact between low and high SES neighborhoods for both the teacher and student perspectives were identified. In multilingual families, however, significant differences were found. Teachers reported more home‐to‐school contact with multilingual families in high SES neighborhoods, whereas students reported more positive contact quality in low SES neighborhoods. The effect sizes for these interactions were, however, rather low.

To test the third hypothesis (H3), a multigroup regression analysis was conducted to examine predictors of students' sense of school belonging, separately in high and low SES neighborhoods (Table 5). Predictors included the perspectives of parents, teachers, and students on home‐to‐school contact, as well as the only‐German‐speaking status of students. In high SES neighborhoods, the regression model was significant, *F*(4,95) = 2.64, *p* = 0.05, and explained 10% of the variance in students' school belonging (*R* ^2^ = 0.10). The results indicated that home‐to‐school contact from the parental and students' perspectives were significant positive predictors of students' sense of school belonging (parents: *β* = 0.245, *p* = 0.04; students: *β* = 0.228, *p* = 0.05), while teachers' perspective was a negative predictor (teachers: *β* = −0.210, *p* = 0.05). The status of being in an only‐German‐speaking family did not significantly predict school belonging in high SES schools (*β* = −0.070, *p* = 0.51).

**Table 5:** Multigroup regression on Students' School Belonging as Dependent Variable.

Variable		High SES neighborhoods				Low SES neighborhoods			
		*β*	SE	*t*	*p*	*β*	SE	*t*	*p*
Constant			0.871	4.324	0.000		2.154	1.142	0.264
Home‐to‐School Contact	Parental Perspective on Contact Frequency	0.245	0.174	2.114	0.04*	0.155	0.198	0.848	0.40
	Teachers’ Perspective on Contact Frequency	−0.210	0.173	−1.827	0.05*	−0.345	0.316	−1.904	0.05*
	Students’ Perspective on Contact Quality	0.228	0.174	1.905	0.05*	0.338	0.190	1.901	0.05*
Only‐German‐speaking Families		−0.070	0.408	−0.665	0.51	−0.567	0.694	−2.588	0.02*
		*F* = 2.64, *p* = 0.05, *R* ^2^ = 0.10				*F* = 2.68, *p* = 0.05, *R* ^2^ = 0.29			

* *p* ≤ 0.05.

In low SES neighborhoods, the regression model was also significant, *F*(4,95) = 2.68, *p* = 0.05, accounting for 29% of the variance in students' school belonging (*R* ^2^ = 0.29). In these schools, home‐to‐school quality of contact from students' perspective was found to be a significant positive predictor, while contact frequency from teachers' perspective was a negative predictor (teachers: *β* = −0.345, *p* = 0.05; students: *β* = 0.338, *p* = 0.05). In contrast, contact frequency from the parental perspective did not significantly predict school belonging (*β* = 0.155, *p* = 0.40). Additionally, being only‐German‐speaking was significantly negatively associated with students' sense of school belonging in low‐SES neighborhoods (β = −0.567, *p* = 0.02). Thus, Hypothesis 3 was partly supported by the data.

## 5 Discussion

The present mixed‐methods study explored the home‐to‐school contact across schools in low and high SES neighborhoods and its association with students' sense of school belonging. The study demonstrated qualitative disparities in the provision of structural support at the institutional level and quantitative discrepancies in individual perceptions regarding home‐to‐school contact at the individual level. The SES status of schools' neighborhoods and the family language seemed to affect the home‐to‐school contact opportunities. In combination, our results show differences between schools in high and low SES neighborhoods on both the structural and individual level, with evident associations on students' reported school belonging. In addition, the study revealed that family language must be considered when differences in home‐to‐school contact and students' school belonging are to be explained.

The qualitative findings on the structural level of home‐to‐school contact revealed differences between schools in low‐ and high‐SES neighborhoods. Schools in low SES neighborhoods treated diversity more often as a static concept, with efforts focused on support for students and parents. These schools also tend to have more formal structures for parent engagement, such as career counseling and extensive parental support services (e.g., financial assistance), which probably reflect the greater needs of these communities. In contrast, schools in high SES neighborhoods approached diversity dynamically, viewing it as a factor that could be leveraged for educational enrichment and personal growth. These schools offered more specialized and diverse engagement opportunities, such as thematic parent‐teacher conferences and online resources, highlighting innovative approaches leveraging their more abundant resources. Nevertheless, there were also commonalities in the practices adopted by schools regardless of neighborhood SES, such as Open House Days or Parents' Councils. In general, all schools showed extensive efforts to engage parents in school activities but obviously developed slightly different contact strategies. Unfortunately, the qualitative data cannot reveal why the strategies differed. On one hand, it may well be the case that schools in different neighborhoods adjust to the varied needs of their parents. On the other hand, the different strategies may be the result of differences in the endowment with resources that allow schools in high SES areas to develop more sophisticated contact strategies. Future research should focus on this question.

Quantitative analyses on the individual level showed significant differences in home‐to‐school contact depending on the family language. Teachers and parents reported more frequent contact in only‐German‐speaking families compared to multilingual families, which is also underscored by the significant differences in language skills: In our sample, multilingual families showed lower levels of German language skills than only‐German‐speaking families. Language barriers can, therefore, hinder effective home‐to‐school contact (Schneider and Arnot 2018). Moreover, analyses revealed an interaction effect of neighborhood SES and family language. From the teachers' perspective, communication with families in lower SES neighborhoods is often perceived as more challenging when families speak other languages than the school's dominant language (Goodall and Montgomery 2014; Turney and Kao 2009). The finding that these schools tend to have a higher percentage of teachers with migration history (Table 1) may indicate that these schools make efforts to provide a more representative teaching staff attuned to their linguistic diverse communities (Guo 2012). Surprisingly, despite the fact that teachers reported less contact with multilingual parents, students perceived this contact as rather positive. One explanation for the students' perspectives is associated with the potential role of students in these contact situations. Students from immigrant background often speak the dominant language better than their parents and serve as language or culture brokers‐ even in second‐generation immigrant families and particularly in the communication with schools (Titzmann 2012; Weisskirch and Alva 2002). In this role, students may have better insights into the home‐to‐school contact and may realize that this communication is in their own interest and, hence, may value this contact although it may occur less frequently as compared to other families. This finding again highlights the active role multilingual students play in navigating their own future.

Our study also explored the impact of home‐to‐school contact on students' sense of school belonging, hypothesizing that increased contact would enhance the connectedness to school. The results highlight distinct patterns in predictors of school belonging between schools in high and low SES neighborhoods. In high SES neighborhoods, parents' contact frequency and students' contact quality reports on home‐to‐school contact positively affected students' sense of belonging, whereas teachers' contact frequency reports had a negative effect. In low SES neighborhoods, the effects for teachers and students reports were the same as in high SES neighborhoods. The parental perspective, however, was not associated with school belonging in these schools. One explanation for the varying perspectives may be that in schools from high SES neighborhoods parents are able to rely on more personal and structural resources and make a difference on students' outcomes, while in low SES neighborhoods where parents dispose of less resources, teachers' role becomes more important and can compensate for some of the missing parental input. As our study suggests, there is a need for more research that explores the teacher and parents' role in different SES neighborhoods. Students' perspective on parent–teacher contact quality was an important predictor in both types of schools indicating that students' perceptions of school belonging are salient across different contexts. Somewhat surprising was the finding that students from only‐German‐speaking families reported lower levels of school belonging, but only in low SES neighborhoods. This finding requires more research, and we can only speculate on this effect. Only‐German‐speaking students may perceive efforts to include multilingual peers as a shift in attention, potentially evoking discomfort or a perceived challenge to their linguistic position. Another possible explanation relates to school selection processes. As previous research has shown, school choice in Germany often involves strategic decision‐making by families aiming to place their children in schools with high academic prospects (Riedel et al. 2010). Consequently, only‐German‐speaking students attending schools in lower SES neighborhoods may disproportionately come from families with fewer resources or different educational priorities, which could be associated with lower levels of school bonding. A third explanation why a diversity‐friendly school climate (e.g., multiple identities, multiple languages) is viewed as a challenge may be that a climate of diversity contrasts the prevailing societal norm to favor assimilation (Schachner et al. 2018; Verkuyten and Thijs 2002). Future research will determine which explanation holds true.

Overall, our findings complement existing research on the impact of SES and linguistic diversity on parental school involvement (Hill and Tyson 2009). One important implication is the necessity for schools to implement diverse and multilingual communication strategies to ensure all families can participate fully in their children's education. Research has also shown that the use of simple language in communicating with parents can be a step forward (Horgan et al. 2022). The differentiated approaches observed in low and high SES neighborhoods also reflect broader socioeconomic disparities that influence resource allocation, communication strategies, and ultimately, student outcomes. Families in higher SES contexts can leverage more resources and networks than families in lower SES contexts, confirming the Matthew Effect in education.

One of the key strengths of this study is the combination of qualitative and quantitative methods, which provides new insights and a comprehensive understanding of the structural and individual levels of home‐to‐school contact. Qualitative data highlighted the structural differences and specific strategies employed by schools, while quantitative analysis allowed us to draw conclusions about mechanisms for interindividual differences in perceptions. In terms of Bronfenbrenner's model (1979), the qualitative and the quantitative part are intertwined because both capture different characteristics of the mesosystems home–school and school‐neighborhood that shape student development. Another strength is the use of multiple informants (classroom teachers, students and their parents) on home‐to‐school contact, which comes with the opportunity to examine matches and mismatches between perspectives on home‐to‐school contact. The distinct findings on the multiple perspectives also point out that home‐to‐school contact is not only a bidirectional process, but a multidirectional process, where contact takes place on different paths: school–home, home–school, or school–home–school.

However, certain limitations of this study could be addressed in future research. For example, future studies should consider conducting in‐depth interviews with the school personnel about the school's diversity‐related code of conduct and communication strategies. Information on the school's homepage offers only one perspective about school life, while different teachers and school staff may have additional or contradicting perspectives. We also do not know to what extent homepage statements are part of schools daily practice in communicating with parents. Another limitation is the study's cross‐sectional design, which constrains the ability to draw causal conclusions about the relationships between SES, language, communication strategies, and student outcomes. Furthermore, the reliance on self‐reported data introduces potential bias, as participants' responses may be shaped by perceptions or the desire to present themselves favorably. Future research would benefit from using longitudinal designs to track changes over time and incorporating objective measures of contact and engagement to complement self‐reports. A final limitation is that we used different instruments to assess home‐to‐school contact–teachers and parents answered the same items on contact frequency, while students reported on the contact quality between their parents and classroom teachers, which does not fully represent a triadic perspective on the same construct. Nevertheless, even the same scales may measure different aspects between different stakeholders as they can focus on different aspects when answering the same items (Paizan et al. 2024).

### 5.1 Practical Implications

Our results suggest several practical implications, particularly in the formulation of educational policies and practices aimed at reducing educational disparities. The differences observed in home‐to‐school contact across SES groups illustrate that education requires more than recognizing diversity—it demands proactive engagement with all students and their families, regardless of their background. Schools must foster environments where parents feel welcomed and valued, thereby promoting a stronger sense of belonging among students. This entails creating easy‐to‐access services for diverse family communities, accommodating different languages, and establishing a strong network of institutions in the long term that supports the community, such as migrant organizations, multicultural professionals and other schools in the area. Especially schools in low SES areas need targeted interventions that facilitate better communication strategies with parents. Translating school information in an easy‐to‐understand version of the main language or preparing teachers and parents to work with AI‐assisted translation software may be first steps to ease the contact between schools and families. Furthermore, offering flexible meeting hours that consider the work schedules of parents in less affluent neighborhoods or changing the communication tools to an online platform are additional solutions to improve home‐to‐school contact. Regarding teachers, there is a need for professional teacher training that enhances their skills in engaging with diverse families. Training should focus on three dimensions (Ehrke et al. 2020): (1) awareness–understanding the socioeconomic challenges faced by families, (2) knowledge—intercultural competence and (3) action – testing and developing effective communication strategies. Overall, our study contributes to the understanding of how structural and individual levels influence home‐to‐school contact and emphasizes the need for context‐specific strategies to promote diverse and supportive school communities.

## 6 Conclusion

Our study provides valuable insights into the complexities of home‐to‐school contact in the German educational context, particularly concerning SES and language. The findings suggest that while schools in high SES neighborhoods exhibit more robust communication strategies, there is a need for targeted support in schools in low SES neighborhoods to bridge the communication gap and reduce educational disparities. Addressing these disparities is crucial to improve home‐to‐school contact on student outcomes and to foster a more equitable educational environment in the long term.

## Conflicts of Interest

The authors declare no conflicts of interest.

## Positionality Statement

Mădălina A. Paizan and Lara Aumann share first authorship. Mădălina A. Paizan is an ethnic minority scholar in Germany, bilingual in Romanian and German. Lara Aumann and Peter F. Titzmann are ethnic white majority scholars from Germany. All authors have expertise in research on diverse youth and directly worked with participating students, teachers, and school principals. While conducting the project, the research team applied different measures (e.g., creating consent forms in different languages, informing participants about their rights in the research process) to support each student, teacher, and parent in the study process and to make participants feel represented and able to express their opinions.

## Data Availability

The data that support the findings of this study are available from the corresponding author upon reasonable request.

## References

[jcop70066-bib-0001] Ahmadi, S. , M. Hassani , and F. Ahmadi . 2020. “Student‐And School‐Level Factors Related to School Belongingness Among High School Students.” International Journal of Adolescence and Youth 25, no. 1: 741–752. 10.1080/02673843.2020.1730200.

[jcop70066-bib-0002] Allen, K. , M. L. Kern , D. Vella‐Brodrick , J. Hattie , and L. Waters . 2018. “What Schools Need to Know about Fostering School Belonging: A Meta‐Analysis.” Educational Psychology Review 30: 1–34. 10.1007/s10648-016-9389-8.

[jcop70066-bib-0004] Barnard, W. M. 2004. “Parent Involvement in Elementary School and Educational Attainment.” Children and Youth Services Review 26, no. 1: 39–62. 10.1016/j.childyouth.2003.11.002.

[jcop70066-bib-0005] Baumert, J. , R. Watermann , and G. Schümer . 2003. “Disparitäten der Bildungsbeteiligung und des Kompetenzerwerbs: Ein institutionelles und individuelles Mediationsmodell [Disparities in Educational Participation and Skills Acquisition: An Institutional and Individual Mediation Model],” Zeitschrift für Erziehungswissenschaft 6: 46–71.

[jcop70066-bib-0006] Bronfenbrenner, U. 1979. “Contexts of Child Rearing: Problems and Prospects.” American Psychologist 34, no. 10: 844–850. 10.1037/0003-066X.34.10.844.

[jcop70066-bib-0007] Cline, T. , S. Crafter , and E. Prokopiou . 2014. “Child Language Brokering in Schools: A Discussion of Selected Findings From a Survey of Teachers and Ex‐Students.” Educational and Child Psychology 31: 33–44. 10.53841/bpsecp.2014.31.2.33.

[jcop70066-bib-0008] Coady, M. R. , and R. Ankeny . 2019. “Engaging Families in the US: Research and Practice for Educators.” LEARN 8: 56–69. https://www.researchgate.net/publication/340062010_Engaging_Multilingual_Families_in_the_US_Research_and_Practice_for_Educators.

[jcop70066-bib-0009] Creswell, J. W. , and V. L. P. Clark . 2017. Designing and Conducting Mixed Methods Research. 2nd ed. SAGE Publications.

[jcop70066-bib-0010] Desforges, C. , and A. Abouchaar . 2003. *The Impact Of Parental Involvement, Parental Support and Family Education on Pupil Achievement and Adjustment: A Literature Review* (Research Report No. 433). Department for Education and Skills. https://library.bsl.org.au/jspui/bitstream/1/3644/1/Impact%20of%20Parental%20Involvement_Desforges.pdf.

[jcop70066-bib-0011] Díez, J. , S. Gatt , and S. Racionero . 2011. “Placing Immigrant and Minority Family and Community Members at the School's Centre: The Role of Community Participation.” European Journal of Education 46, no. 2: 184–196. 10.1111/j.1465-3435.2011.01474.x.

[jcop70066-bib-0012] Ehrke, F. , A. Ashoee , M. C. Steffens , and E. Louvet . 2020. “A Brief Diversity Training: Raising Awareness of Ingroup Privilege to Improve Attitudes Towards Disadvantaged Outgroups.” International Journal of Psychology 55, no. 5: 732–742. 10.1002/ijop.12665. 32080847

[jcop70066-bib-0013] Epstein, J. L. , and M. G. Sanders . 2002. “Family, School, and Community Partnerships.” In Handbook of Parenting Volume 5 Practical Issues in Parenting, edited by M. C. Bornstein , 2nd ed., 407–437. Lawrence Erlbaum Associates.

[jcop70066-bib-0014] Garbacz, S. A. , R. T. Santiago , D. Kosty , et al. 2021. “Examining Congruence in Parent–Teacher Perceptions of Middle School Supports for Students and Families.” Psychology in the Schools 58, no. 6: 1169–1184. 10.1002/pits.22495.

[jcop70066-bib-0015] Garbacz, S. A. , A. A. Zerr , T. J. Dishion , J. R. Seeley , and E. Stormshak . 2018. “Parent Educational Involvement in Middle School: Longitudinal Influences on Student Outcomes.” Journal of Early Adolescence 38, no. 5: 629–660. 10.1177/0272431616687670. 29731534 PMC5931399

[jcop70066-bib-0016] Gibbs, B. G. , M. Marsala , A. Gibby , et al. 2021. “Involved Is an Interesting Word”: An Empirical Case for Redefining School‐Based Parental Involvement as Parental Efficacy.” Social Sciences 10, no. 5: 156. 10.3390/socsci10050156.

[jcop70066-bib-0017] Goodall, J. , and C. Montgomery . 2014. “Parental Involvement to Parental Engagement: A Continuum.” Educational Review 66, no. 4: 399–410. 10.1080/00131911.2013.781576.

[jcop70066-bib-0018] Goodenow, C. 1993. “The Psychological Sense of School Membership Among Adolescents: Scale Development and Educational Correlates.” Psychology in the Schools 30, no. 1: 79–90.

[jcop70066-bib-0019] Guo, Y. 2012. “Diversity in Public Education: Acknowledging Immigrant Parent Knowledge.” Canadian Journal of Education 35, no. 2: 120–140.

[jcop70066-bib-0020] Haines, S. J. , J. M. S. Gross , M. Blue‐Banning , G. L. Francis , and A. P. Turnbull . 2015. “Fostering Family–School and Community–School Partnerships in Inclusive Schools: Using Practice as A Guide.” Research and Practice for Persons With Severe Disabilities 40, no. 3: 227–239. 10.1177/1540796915594141.

[jcop70066-bib-0021] Hébert, C. , J. Goulet , K. Tardif‐Grenier , L. S. Pagani , and I. Archambault . 2024. “Links Between Family‐School Value Discrepancies and Teacher‐Student Relations in Adolescents From Immigrant and Nonimmigrant Backgrounds.” Journal of Education 204, no. 4: 691–702. 10.1177/00220574241235684.

[jcop70066-bib-0022] Hill, N. E. , and D. F. Tyson . 2009. “Parental Involvement in Middle School: A Meta‐Analytic Assessment of the Strategies That Promote Achievement.” Developmental Psychology 45, no. 3: 740–763. 10.1037/a0015362. 19413429 PMC2782391

[jcop70066-bib-0023] Horgan, D. , S. Martin , J. O'Riordan , and R. Maier . 2022. “Supporting Languages: The Socio‐Educational Integration of Migrant and Refugee Children and Young People.” Children & Society 36: 369–385. 10.1111/chso.12525.

[jcop70066-bib-0064] Hußmann, A. , H. Wendt, W. Bos, et al. 2017. IGLU 2016. Lesekompetenzen von Grundschulkindern in Deutschland im internationalen Vergleich [Reading Skills of Primary School Children in Germany in International Comparison]. Waxmann. 10.25656/01:15476.

[jcop70066-bib-0025] KMK . 2018. Bildung und Erziehung als gemeinsame Aufgabe von Eltern und Schule. Beschluss der Kultusministerkonferenz vom 11.10.2018 in der Fassung vom 23.06.2022 [Learning and Education as a Joint Responsibility of Parents and Schools. Resolution of the Conference of Ministers of Education and Cultural Affairs of 11 October 2018, as Amended on 23 June 2022]. https://www.kmk.org/fileadmin/Dateien/veroeffentlichungen_beschluesse/2018/2018_10_11-Dokumentation-Bildung-und-Erziehung.pdf.

[jcop70066-bib-0026] Kohl, K. , J. Jäkel , O. Spiegler , J. A. Willard , and B. Leyendecker . 2013. “Eltern und Schule‐Wie Beurteilen Türkischstämmige Und Deutsche Mütter Sowie Deutsche Lehrkräfte Elterliche Verantwortung Und Beteiligung? [Parents and School – How do Mothers of Turkish Origin and German Mothers, as Well as German Teachers, Assess Parental Responsibility and Involvement?],” Psychologie in Erziehung und Unterricht 61, no. 2: 96–111. 10.2378/peu2013.art21d.

[jcop70066-bib-0027] Korpershoek, H. , E. T. Canrinus , M. Fokkens‐Bruinsma , and H. De Boer . 2020. “The Relationships Between School Belonging and Students' Motivational, Social‐Emotional, Behavioural, and Academic Outcomes in Secondary Education: A Meta‐Analytic Review.” Research Papers in Education 35, no. 6: 641–680. 10.1080/02671522.2019.1615116.

[jcop70066-bib-0065] Landeshauptstadt Hannover, Dezernat für Soziales und Integration. 2024. Sozialbericht 2023. Soziale Entwicklungen in der Einwanderungsstadt Hannover. Teilhabe, Zugangschancen & kommunale Handlungsspielräume [Social Report 2023. Social Developments in the Immigrant City of Hanover. Participation, Access Opportunities & Municipal Scope for Action] Hannover.

[jcop70066-bib-0028] Lasater, K. 2016. “Parent‐Teacher Conflict Related to Student Abilities: The Impact on Students and the Family‐School Partnership.” School Community Journal 26, no. 2: 237–262.

[jcop70066-bib-0029] Leithwood, K. 2021. “A Review of Evidence About Equitable School Leadership.” Education Sciences 11, no. 8: 377. 10.3390/educsci11080377.

[jcop70066-bib-0030] Mayring, P. 2003. Qualitative Inhaltsanalyse‐Grundlagen und Techniken [Qualitative Content Analysis‐Basics and Methods], 6th ed. Beltz.

[jcop70066-bib-0031] McWayne, C. M. , G. Melzi , and J. Mistry . 2022. “A Home‐to‐School Approach for Promoting Culturally Inclusive Family–School Partnership Research and Practice.” Educational Psychologist 57, no. 4: 238–251. 10.1080/00461520.2022.2070752.

[jcop70066-bib-0032] Moral, C. , L. Higueras‐Rodríguez , A. Martín‐Romera , E. M. Valdivia , and A. Morales‐Ocaña . 2020. “Effective Practices in Leadership for Social Justice. Evolution of Successful Secondary School Principalship in Disadvantaged Contexts.” International Journal of Leadership in Education 23, no. 2: 107–130. 10.1080/13603124.2018.1562100.

[jcop70066-bib-0033] Munthe, E. , and E. Westergård . 2023. “Parents', Teachers', and Students' Roles in Parent‐Teacher Conferences; a Systematic Review and Meta‐Synthesis.” Teaching and Teacher Education 136: 104355. 10.1016/j.tate.2023.104355.

[jcop70066-bib-0034] Murray, D. W. , D. Rabiner , A. Schulte , and K. Newitt . 2008. “Feasibility and Integrity of a Parent–Teacher Consultation Intervention for ADHD Students.” In Child & Youth Care Forum, Vol. 37, 111–126. Springer US. 10.1007/s10566-008-9054-6.

[jcop70066-bib-0035] Murray, E. , L. McFarland‐Piazza , and L. J. Harrison . 2015. “Changing Patterns of Parent–Teacher Communication and Parent Involvement From Preschool to School.” Early Child Development and Care 185, no. 7: 1031–1052. 10.1080/03004430.2014.975223.

[jcop70066-bib-0036] Oppermann, E. , and R. Lazarides . 2023. “The Interplay of Gender With Social and Migrant Background in the Development of Elementary School Students' Interest in Mathematics and Language Arts.” Learning and Individual Differences 106: 102324. 10.1016/j.lindif.2023.102324.

[jcop70066-bib-0037] Paizan, M. A. , A. E. F. Benbow , L. Aumann , and P. F. Titzmann . 2022. “Home‐Learning During COVID‐19: The Psychological Adjustment of Minority and Majority Adolescents.” School Psychology 37, no. 1: 75–84. 10.1037/spq0000489. 34928642

[jcop70066-bib-0038] Paizan, M. A. , A. E. F. Benbow , and P. F. Titzmann . 2024. “Relationship Quality in Student‐Teacher‐Dyads: Comparing Student and Teacher Determinants in Multicultural Classrooms.” International Journal of Intercultural Relations 101: 102006. 10.1016/j.ijintrel.2024.102006.

[jcop70066-bib-0039] Park, S. , and S. Holloway . 2018. “Parental Involvement in Adolescents' Education: An Examination of The Interplay Among School Factors, Parental Role Construction, and Family Income.” School Community Journal 28, no. 1: 9–36.

[jcop70066-bib-0040] Pfost, M. , J. Hattie , T. Dörfler , and C. Artelt . 2014. “Individual Differences in Reading Development: A Review of 25 Years of Empirical Research on Matthew Effects in Reading.” Review of Educational Research 84, no. 2: 203–244. 10.3102/0034654313509492.

[jcop70066-bib-0041] Piller, I. , A. S. Bruzon , and H. Torsh . 2023. “Monolingual School Websites as Barriers to Parent Engagement.” Language and Education 37, no. 3: 328–345. 10.1080/09500782.2021.2010744.

[jcop70066-bib-0042] Riedel, A. , K. Schneider , C. Schuchart , and H. Weishaupt . 2010. “School Choice in German Primary Schools. How Binding Are School Districts?” Journal for Educational Research Online 2, no. 1: 94–120.

[jcop70066-bib-0043] Schachner, M. K. , L. Juang , U. Moffitt , and F. J. R. van de Vijver . 2018. “Schools as Acculturative and Developmental Contexts for Youth of Immigrant and Refugee Background.” European Psychologist 23, no. 1: 44–56. 10.1027/1016-9040/a000312.

[jcop70066-bib-0044] Schneider, C. , and M. Arnot . 2018. “Transactional School‐Home‐School Communication: Addressing the Mismatches Between Migrant Parents' and Teachers' Views of Parental Knowledge, Engagement and the Barriers to Engagement.” Teaching and Teacher Education 75: 10–20. 10.1016/j.tate.2018.05.005.

[jcop70066-bib-0045] Schofield, J. W. 2006. Migration Background, Minority‐Group Membership And Academic Achievement: Research Evidence From Social, Educational and Developmental Psychology . AKI.

[jcop70066-bib-0046] Serpell, Z. N. , and A. J. Mashburn . 2012. “Family‐School Connectedness and Children's Early Social Development.” Social Development 21, no. 1: 21–46. 10.1111/j.1467-9507.2011.00623.x.

[jcop70066-bib-0047] Sime, D. , and M. Sheridan . 2014. “‘You Want the Best for Your Kids': Improving Educational Outcomes for Children Living in Poverty Through Parental Engagement.” Educational Research 56, no. 3: 327–342. 10.1080/00131881.2014.934556.

[jcop70066-bib-0048] Skopek, J. , and G. Passaretta . 2021. “Socioeconomic Inequality in Children's Achievement From Infancy to Adolescence: The Case of Germany.” Social Forces 100, no. 1: 86–112. 10.1093/sf/soaa093.

[jcop70066-bib-0049] Smith, T. E. , W. M. Reinke , K. C. Herman , and F. Huang . 2019. “Understanding Family–School Engagement Across and Within Elementary‐And Middle‐School Contexts.” School Psychology 34, no. 4: 363–375. 10.1037/spq0000290. 31294594

[jcop70066-bib-0050] Smith, T. E. , S. M. Sheridan , E. M. Kim , S. Park , and S. N. Beretvas . 2020. “The Effects of Family‐School Partnership Interventions on Academic and Social‐Emotional Functioning: A Meta‐Analysis Exploring What Works for Whom.” Educational Psychology Review 32: 511–544. 10.1007/s10648-019-09509-w.

[jcop70066-bib-0051] Statistisches Bundesamt . 2024. Bevölkerung und Migration: Wanderungen 2023 [Population and Migration: Migration in 2023]. Statistisches Bundesamt. https://www.destatis.de.

[jcop70066-bib-0052] Thomas, V. , J. Muls , F. De Backer , and K. Lombaerts . 2020. “Middle School Student and Parent Perceptions of Parental Involvement: Unravelling the Associations With School Achievement and Wellbeing.” Educational Studies 46, no. 4: 404–421. 10.1080/15295192.2019.1694836.

[jcop70066-bib-0053] Tillery, A. D. , K. Varjas , A. T. Roach , G. P. Kuperminc , and J. Meyers . 2013. “The Importance of Adult Connections in Adolescents' Sense of School Belonging: Implications for Schools and Practitioners.” Journal of School Violence 12, no. 2: 134–155. 10.1080/15388220.2012.762518.

[jcop70066-bib-0054] Titzmann, P. F. 2012. “Growing Up Too Soon? Parentification Among Immigrant and Native Adolescents in Germany.” Journal of Youth and Adolescence 41, no. 7: 880–893. 10.1007/s10964-011-9711-1. 21879381

[jcop70066-bib-0055] Topor, D. R. , S. P. Keane , T. L. Shelton , and S. D. Calkins . 2010. “Parent Involvement and Student Academic Performance: A Multiple Mediational Analysis.” Journal of Prevention and Intervention in the Community 38, no. 3: 183–197. 10.1080/10852352.2010.486297. 20603757 PMC3020099

[jcop70066-bib-0056] Turney, K. , and G. Kao . 2009. “Barriers to School Involvement: Are Immigrant Parents Disadvantaged?.” Journal of Educational Research 102, no. 4: 257–271. 10.3200/JOER.102.4.257-271.

[jcop70066-bib-0057] Ulbricht, J. , M. K. Schachner , S. Civitillo , and P. Noack . 2022. “Teachers' Acculturation in Culturally Diverse Schools‐How Is the Perceived Diversity Climate Linked to Intercultural Self‐Efficacy?” Frontiers in Psychology 13: 953068. 10.3389/fpsyg.2022.953068. 36337492 PMC9634156

[jcop70066-bib-0058] Uslu, F. , and S. Gizir . 2017. “School Belonging of Adolescents: The Role of Teacher‐Student Relationships, Peer Relationships and Family Involvement.” Educational Sciences‐Theory and Practice 17, no. 1. 10.12738/estp.2017.1.0104.

[jcop70066-bib-0059] van de Vijver, F. J. R. , and Y. H. Poortinga . 2002. “Structural Equivalence in Multilevel Research.” Journal of Cross‐Cultural Psychology 33, no. 2: 141–156. 10.1177/0022022102033002002.

[jcop70066-bib-0060] Vaz, S. , M. Falkmer , M. Ciccarelli , et al. 2015. “Belongingness in Early Secondary School: Key Factors That Primary and Secondary Schools Need to Consider.” PLoS One 10, no. 9: e0136053. 10.1371/journal.pone.0136053. 26372554 PMC4570666

[jcop70066-bib-0061] Verkuyten, M. , and J. Thijs . 2002. “Multiculturalism Among Minority and Majority Adolescents in The Netherlands.” International Journal of Intercultural Relations 26, no. 1: 91–108.

[jcop70066-bib-0062] Waanders, C. , J. L. Mendez , and J. T. Downer . 2007. “Parent Characteristics, Economic Stress and Neighborhood Context as Predictors of Parent Involvement in Preschool Children's Education.” Journal of School Psychology 45, no. 6: 619–636. 10.1016/j.jsp.2007.07.003.

[jcop70066-bib-0063] Weisskirch, R. S. , and S. A. Alva . 2002. “Language Brokering and the Acculturation of Latino Children.” Hispanic Journal of Behavioral Sciences 24, no. 3: 369–378. 10.1177/0739986302024003007.

